# Murine amniotic fluid stem cells contribute mesenchymal but not epithelial components to reconstituted mammary ducts

**DOI:** 10.1186/scrt20

**Published:** 2010-07-07

**Authors:** Petra AB Klemmt, Vida Vafaizadeh, Bernd Groner

**Affiliations:** 1 Georg-Speyer-Haus, Institute for Biomedical Research, Paul-Ehrlich-Str. 42-44, 60596 Frankfurt, Germany

## Abstract

**Introduction:**

Amniotic fluid harbors cells indicative of all three germ layers, and pluripotent fetal amniotic fluid stem cells (AFSs) are considered potentially valuable for applications in cellular therapy and tissue engineering. We investigated whether it is possible to direct the cell fate of AFSs *in vivo *by transplantation experiments into a particular microenvironment, the mammary fat pad. This microenvironment provides the prerequisites to study stem cell function and the communication between mesenchymal and epithelial cells. On clearance of the endogenous epithelium, the ductal tree can be reconstituted by the transfer of exogenously provided mammary stem cells. Analogously, exogenously provided stem cells from other tissues can be investigated for their potential to contribute to mammary gland regeneration.

**Methods:**

We derived pluripotent murine AFSs, measured the expression of stem cell markers, and confirmed their *in vitro *differentiation potential. AFSs were transplanted into cleared and non cleared fat pads of immunocompromised mice to evaluate their ability to assume particular cell fates under the instructive conditions of the fat-pad microenvironment and the hormonal stimulation during pregnancy.

**Results:**

Transplantation of AFSs into cleared fat pads alone or in the presence of exogenous mammary epithelial cells caused their differentiation into stroma and adipocytes and replaced endogenous mesenchymal components surrounding the ducts in co-transplantation experiments. Similarly, transplantation of AFSs into fat pads that had not been previously cleared led to AFS-derived stromal cells surrounding the elongating endogenous ducts. AFSs expressed the marker protein *α-SMA*, but did not integrate into the myoepithelial cell layer of the ducts in virgin mice. With pregnancy, a small number of AFS-derived cells were present in acinar structures.

**Conclusions:**

Our data demonstrate that the microenvironmental cues of the mammary fat pad cause AFSs to participate in mammary gland regeneration by providing mesenchymal components to emerging glandular structures, but do not incorporate or differentiate into ductal epithelial cells.

## Introduction

The developing fetus is surrounded by a protective layer of liquid, the amniotic fluid. This fluid provides mechanical protection as well as nutrients required for fetal growth and well-being and contains cells derived from embryonic and extraembryonic tissues [1]. The initial cultures of amniotic fluid cells comprise a heterogeneous mixture of cell types [2,3], and differentiated cells, progenitor cells, and embryonic-like stem cells have been detected [4]. These cells exhibit differences in their adherence to culture plates. During prolonged culture, these cells acquire a more-homogeneous appearance and resemble fibroblast-like cells. The different origins of these cells can be determined with immunohistochemistry. The majority of amniotic fluid cells appear fibroblastoid, are rapidly proliferating, and co-express keratins and vimentin [5-7]. Cells derived from backflush cultures of amniocentesis specimens, obtained for prenatal genetic-screening procedures, were used for *in vitro *differentiation assays. They initially demonstrated multipotency, and the expression of embryonic stem cell markers suggested the presence of a pluripotent subpopulation [4,8,9].

In the meantime, it has been shown that amniotic fluid-derived stem cells (AFSs) are able to differentiate into neurogenic lineages and thus contribute to the ectodermal layer; into osteoblasts, fibroblasts, adipocytes, chondrocytes, and endothelial cells, as part of the mesodermal lineage, and into hepatocytic cells as part of the endodermal lineage. These cell-differentiation programs were triggered by culturing the AFSs in a wide spectrum of different media [10]; for example, basic fibroblast growth factor (bFGF), epidermal growth factor (EGF), and *N*-ethylmaleimide-sensitive factor (NSF-1) were required to induce the neuronal differentiation [11], and indomethacin, dexamethasone, methyl-3-isobutylxanthine, and insulin were added to the medium to achieve adipogenesis [8]. The multipotent nature of AFSs and the possibilities of differentiating them into diverse cell types have made them interesting candidates for therapeutic applications.

We investigated the plasticity of murine AFSs and their potential to adapt to a particular microenvironment. For this purpose, we performed transplantation experiments of AFSs into the mammary fat pads of mice.

The mammary gland is a highly dynamic organ; its development is mainly postnatal, and its tissue composition changes in a characteristic fashion during puberty, pregnancy, lactation, and involution [12]. The branching ducts are of ectodermal origin and consist of a single layer of luminal epithelial cells surrounded by a contractile layer of myoepithelial cells, causing milk ejection with oxytocin induction. The myoepithelial cells are in direct contact with an extracellular basement membrane and contribute its components. They are instrumental for the establishment of ductal polarity and lumen formation [13]. The mammary ducts are embedded in an adipose stroma of mesodermal origin, which consists mainly of adipocytes, but also comprises fibroblasts, endothelial cells, and immune cells involved in the regulation of mammogenesis [14]. The ducts infiltrate the adipose stroma during puberty, and the alveolar structures that grow out during pregnancy are interspersed with islands of adipocytes. During lactation, the adipocytes lose most of their fat content, but persist as long projections in the interstitial space. A layer of fibrous connective tissue is present around the ducts and the secretory alveoli [15].

The cyclical nature of cell proliferation, differentiation, and apoptosis and the regenerative capacity of the epithelium during successive cycles of pregnancy, lactation, and involution require the presence and activity of stem cells [16,17]. A unique feature of mammary stem cells is their ability to reconstitute the ductal component of the mammary gland with transplantation into cleared fat pads [18]. This is a distinct property of the mammary system and is comparable to the reconstitution of the hematopoietic system by stem cell transplantation [19]. In these circumstances, stem cells are functionally selected by their reconstitution ability *in vivo*.

Transplantation of stem cells from adult male seminiferous tubules and of neural stem cells from embryonic or adult brain into fat pads was shown to adopt functional mammary epithelial cell traits, but only in conjunction with normal, stem cell-depleted mammary epithelial cells after pregnancy. These results indicate that tissue-specific signals from the mammary stroma cooperate with signals originating from differentiated mammary epithelial cells and that these combined influences are capable of directing the fate of stem cells from heterologous organs [20,21].

The ready availability of AFSs and the expectations that these cells might become a convenient source for therapeutic applications caused us to investigate their adaptability to a particular organ microenvironment and their potential to contribute to mammary gland structures. Cultured AFSs by themselves or mixed with primary mammary epithelial cells were introduced into cleared and noncleared fat pads of mice to gauge their plasticity toward mammary epithelial differentiation. Our results indicate that the signals operating in this microenvironment trigger an exclusive mesenchymal differentiation program of transplanted AFSs, irrespective of the presence of mammary epithelial cells in virgin mice. The AFSs proliferate and persist during mammary gland development, interact closely with endogenous or transplanted mammary epithelial cells, and provide stromal components and adipocytes surrounding the ductal structures. Under the hormonal influence of pregnancy, a very small number of AFS-derived cells appear present in acinar structures between the basement membrane and the myoepithelium.

## Materials and methods

### Mice

C57BL/6-Tg (ACTB-EGFP) and B6;129S-*GT(ROSA)26Sor*/J (Rosa26) from Jackson Laboratories (Charles River, Sulzfeld, Germany) were used as donor mice for amniotic fluid or mammary epithelial cell isolation. Female RAG2 KO mice (BALB/cA-RAG2KO, IL-2RγKO) from Central Institute for Experimental Animals (CIEA, Kawasaki, Japan) were used as hosts for the transplantation studies. All mice were maintained in a pathogen-free facility, and the experimental procedures were approved by the Animal Welfare office of the Regierungspräsidium Darmstadt.

### Isolation and culture of AFSs from murine amniotic fluid

Amniotic fluid was collected from pregnant donor mice at embryonic day 13.5 by rupturing the yolk sac with a 27-gauge needle, and recovered cells were cultured in 35-mm dishes in AmnioMax complete (Invitrogen, Darmstadt, Germany) by using a two-stage culture method [9]. In brief, after an initial expansion of 5 days, the nonadherent amniotic fluid cells in the supernatant were collected, centrifuged for 5 min at 300 *g*, and replated in AmnioMax complete for a further 5 days, by which they acquired an adherent phenotype. Established AFS lines were passaged at a dilution of 1:4 to 1:8 in DMEM supplemented with 10% fetal calf serum (FCS), 50 units/ml and 50 μ/ml penicillin-streptomycin, and 2 m*M *L-glutamine (DMEM complete; all from Invitrogen, Darmstadt, Germany).

### FACS analysis

For flow-cytometric analysis, 5 × 10^5 ^cells were fixed for 5 min in 3% para-formaldehyde (PFA, Sigma-Aldrich, Munich, Germany), washed with PBS (PAA Laboratories GmbH, Cölbe, Germany), and incubated with 1 μl fluorescence-conjugated antibodies in 200 μl PBS against *CD24*, *CD29*, *CD44*, *CD117*, *Sca-1*, *CD49f*, *CD90.2 *(all BD Biosciences, Heidelberg, Germany), and *CD31 *and *CD45 *(Biolegend, BIOZOL Diagnostica Vertrieb GmbH, Eching, Germany) or their respective IgG controls for 20 min in the dark. After two washing steps with PBS, cells were analyzed by using a FACS Scan (Becton Dickinson, Heidelberg, Germany) flow cytometer with CellQuest software (Becton Dickinson, Heidelberg, Germany).

### Immunocytochemistry

AFS cultures were fixed in 3% PFA, permeabilized (10 m*M *HEPES, 200 m*M *sucrose, 3 m*M *MgCl_2_, 50 m*M *NaCl, 0.5% Triton X-100, 0.2% NaN_3_, all from Sigma-Aldrich, Munich, Germany) and blocked in 3% BSA (Sigma-Aldrich, Munich, Germany). Specific antigens were detected by incubation with antibodies to α-*keratin 18 *(clone KS18.04; Progen Biotechnik GmbH, Heidelberg, Germany, ready-to-use), α-*keratin 14 *(clone AF64; Covance, HiSS Diagnostics GmbH, Germany, 1:200), α-*vimentin *(ab45939; abCam, Cambridge, UK, 1:500), α-*CD44*-CyChrome (clone IM7; BD Biosciences, Heidelberg, Germany, 1:60), and α-*SMA *(clone 1A4; Sigma-Aldrich, Munich, Germany, 1:500) for 1 h at RT in 0.1% BSA followed by incubation with 10 μ/ml of donkey anti-mouse AlexaFluor546-conjugated IgG (Invitrogen, Darmstadt, Germany) or donkey anti-rabbit AlexaFluor488-conjugated IgG (Invitrogen, Darmstadt, Germany) for 45 min at RT in the dark and mounted in Mowiol containing 1.5 μg/ml DAPI. Staining was assessed by using a Nikon Eclipse TE300 microscope and NIS Elements AR imaging software (Nikon, Düsseldorf, Germany).

### Differentiation protocols

AFS were cultured to confluence in DMEM complete before the addition of adipogenic supplements (1 μ*M *dexamethasone, 0.5 m*M *isobutylmethylxanthine, 5 μ/ml Insulin, and 60 μ*M *indomethacin, all from Sigma-Aldrich, Munich, Germany), osteogenic supplements (100 n*M *dexamethasone, 10 m*M *β-glycerolphosphate, and 50 μ*M *2-phospho ascorbic acid, all from Sigma-Aldrich, Munich, Germany) or epithelial supplements (10 ng/ml keratinocyte growth factor, 10 ng/ml hepatocyte growth factor, 60 ng/ml insulin-like growth factor II (all from BIOZOL Diagnostica Vertrieb GmbH, Eching, Germany), and20 ng/ml epidermal growth factor (BD Biosciences, Heidelberg, Germany),) in DMEM containing 10% FCS and antibiotics for 14 days. The differentiation medium was changed every 4 days. The differentiation potential for adipogenesis was assessed with the visualization of lipid vacuoles by using Oil Red O staining (Sigma-Aldrich, Munich, Germany); the deposition of mineralized calcium accumulation during osteogenesis was detected by Alizarin Red S staining (Sigma-Aldrich, Munich, Germany), and the epithelial character was confirmed with Ayoub Shklar staining [22].

### Measurement of mRNA expression with RT-PCR

Total RNA was extracted from cultured, noninduced or differentiated AFSs by using NucleoSpin RNA II (Macherey & Nagel, Düren, Germany) according to the manufacturer's instructions. RNA was then reverse transcribed by using Superscript II reverse transcriptase (Invitrogen, Darmstadt, Germany), and RT-PCR was performed by using standard protocols. The amplified DNA fragments were subjected to 1.5% agarose gel electrophoresis.

### Isolation and culture of mammary epithelial cells

Mammary glands were isolated from 10-week-old virgin female C57BL/6-Tg (ACTB-EGFP) or B6;129S-*GT(ROSA)26Sor*/J, mice as previously described [17]. The organoids were purified with pulse centrifugation and plated on a thin gel of collagen type I (BD Biosciences, Heidelberg, Germany) in Ham's F-12 (PAA Laboratories GmbH, Cölbe, Germany) supplemented with 5% FCS, 5 μ/ml insulin (Sigma-Aldrich, Munich, Germany), 1 μ/ml hydrocortisone (Sigma-Aldrich, Munich, Germany), 5 ng/ml EGF, 50 units/ml-50 μ/ml penicillin-streptomycin, and 50 μ/ml gentamicin (Invitrogen, Darmstadt, Germany). Primary cultures were passaged once, 3 to 4 days after plating before transplantation.

### Three-dimensional cell-culture system

The 3D culture of AFSs and MECs was performed as described [23]. In brief, cells were trypsinized and resuspended in assay medium (DMEM/F12 containing 2% FCS, 0.5 μ/ml hydrocortisone, 100 ng/ml choleratoxin (Sigma-Aldrich, Munich, Germany), 10 μ/ml insulin) at a concentration of 25,000 cells/ml. Eight-chamber glass slides (Nunc, VWR International GmbH, Darmstadt, Germany) were coated with 45 μl growth factor reduced Matrigel (BD Biosciences, Heidelberg, Germany) per well and left to gel at 37°C for 15 min. The cells were mixed 1:1 with assay medium supplemented with 4% Matrigel and 10 ng/ml EGF, and 400 μl of this mixture was added to each well. Assay medium supplemented with 2% Matrigel and 5 ng/ml EGF was replaced every 4 days. Acini formation was assessed after 5 days by using a Nikon Eclipse TE300 microscope and NIS Elements AR imaging software (Nikon, Düsseldorf, Germany) and after 10 days by using a Leica TCS SL confocal microscope and Leica Confocal software version 2.61 (Leica Microsystems, Wetzlar, Germany). Immunostaining of 3D cultures was performed as described [24] with antibodies to α-*vimentin *(ab45939, abCam, Cambridge, UK; 1:500) and *α-SMA *(clone 1A4, Sigma-Aldrich, Munich, Germany; 1:500).

### Cell transplantation into mammary fat pads

The 3-week-old Rag 2^-/- ^recipient mice were anesthetized, and the endogenous epithelium was removed from both number 4 inguinal mammary glands. AFSs alone or mixed with MECs were resuspended in 10 μl cold Matrigel and injected into the remaining fat pad, cleared of its epithelial component. The hosts were analyzed 8 weeks later. The GFP^+ ^outgrowths were visualized by using a Leica MZ16F stereomicroscope and LAS version 2.8 (Leica Microsystems, Wetzlar, Germany), fixed in 4% formalin for 1 h at 4°C, and incubated in X-gal staining solution (10 m*M *K_3_Fe(CN)_6_, 10 m*M *K_4_Fe(CN)_6_, 0.2% NP-40, 0.1% Triton X-100, and 1 mg/ml X-Gal, all from Sigma-Aldrich, Munich, Germany) overnight at 37°C to visualize β-galactosidase-positive outgrowth. Stained glands were rinsed in PBS, photographed, postfixed in 4% formalin, and embedded in paraffin.

### Immunohistochemistry

Immunohistochemical analysis was performed on formalin-fixed paraffin sections (5 μm), cleared in xylene, and rehydrated through an alcohol series. Antigen retrieval by using 10 m*M *sodium citrate buffer, pH 6, was performed for all antibodies except *α-SMA*. IHC-HRP staining was performed by using the Envision detection system Peroxidase/DAB (Dako, Hamburg, Germany) and the M.O.M. kit (Vector Laboratories, BIOZOL Diagnostica Vertrieb GmbH, Eching, Germany) according to the manufacturer's instructions. Slides were counterstained with hematoxylin or Nuclear fast red, dehydrated, and mounted with Assistent-Histokitt. Staining was assessed by using a Leica DMLS microscope (Leica Microsystems, Wetzlar, Germany) and LAS version 2.8. For IHC-IF staining, the sections were blocked in 0.1 mg/ml goat anti-mouse IgG (Jackson ImmunoResearch, Dianova, Hamburg, Germany) and 10% FCS in PBS for 1 h. Primary antibodies were incubated in 1% FCS overnight at 4°C followed by incubation with 10 μ/ml goat anti-mouse AlexaFluor488-conjugated IgG (Invitrogen, Darmstadt, Germany), donkey anti-rabbit AlexaFluor647-conjugated IgG (Invitrogen, Darmstadt, Germany), and donkey anti-goat AlexaFluor594-conjugated IgG (Invitrogen, Darmstadt, Germany) for 45 min at RT in the dark and mounted in Mowiol containing 1.5 μg/ml DAPI. Staining was assessed by using a Leica TCS SP5 confocal microscope and LAS AF imaging software (Leica Microsystems, Wetzlar, Germany). Images were processed by using Imaris (Bitplane, Zurich, Switzerland). The sources of antibodies used in this study: α-GFP (2555; Cell Signaling, New England Biolabs GmbH, Frankfurt, Germany, 1:800); α-*Keratin14 *(clone AF64; Covance, HiSS Diagnostics GmbH, Germany, 1:500); α-*SMA *(clone 1A4; Sigma, Munich, Germany, 1:500), α-*vimentin *(clone C20; Santa Cruz, Heidelberg, Germany, 1:500), α-*c/EBPα *(clone 14AA; Santa Cruz, Heidelberg, Germany, 1:50), and α-*laminin β1 *(clone C19; Santa Cruz, Heidelberg, Germany, 1:50).

## Results

### Amniotic fluid-derived cells exhibit an epithelial phenotype and are multipotent

**Figure 1:**
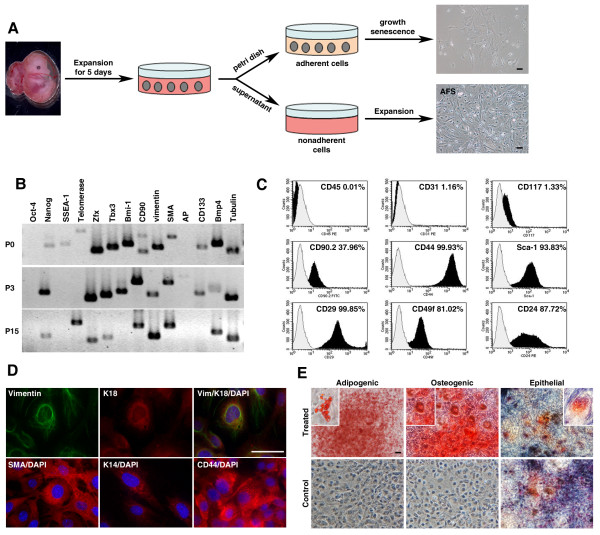
**Isolation, characterization, and differentiation potential of fetal stem cells derived from murine amniotic fluid**. **(a) **Amniotic fluid stem cells (AFSs) were isolated at E13.5 and obtained by using a two-step culture model [9] from C57/B6-TG (ACTB-EGFP) and Rosa26 mice. **(b) **The freshly isolated and cultured AFSs exhibit pluripotency markers and exhibit no spontaneous differentiation in culture. **(c) **Histogram analysis of cell-surface markers showed that AFSs express embryonic and mesenchymal stem cell markers but lack the expression of hematopoietic cell markers. Grey line, isotype control. **(d) **Cultured AFSs exhibit an epithelial phenotype, as demonstrated by the co-expression of keratins and vimentin. **(e) **Differentiation of AFSs into mesodermal cell types after specific stimulation for 14 days is marked by the appearance of lipid granules (adipogenic) or mineralized matrix (osteogenic). The epithelial-stimulation protocol further potentiates the epithelial nature of AFSs (epithelial). Insets show higher magnification of the treated cells. Scale bars, 50 μm.

We collected amniotic fluid from the yolk sac of mice at day 13.5 of pregnancy and isolated amniotic fluid stem cells (AFSs) by using a two-stage culture model [9]. Nonadherent amniotic fluid cells after 5 days of culture were recovered from the medium and replated for a further 5 days (Figure 1a). During this period, they became adherent. In total, we established 20 AFS lines from different donors. The rapidly adhering cells isolated during the initial plating period of 5 days underwent growth senescence within three passages. Morphologically, undifferentiated AFSs obtained during the second culture period appear spindle shaped and resemble fibroblasts.

We characterized the freshly isolated and cultured AFSs with respect to the expression of genes that are indicative of stem-cell phenotypes and measured the mRNA levels of *Oct-4, SSEA-1, Nanog, ZFX, Tbx3*, and *Bmi-1*. These genes have been found to maintain cells in a pluripotent, undifferentiated state, and *Nanog *is a master regulator of pluripotency in embryonic stem cells. The expression of *Nanog, Zfx*, and *Tbx3 *persists at least for 15 passages, and *Bmi-1 *was detected up to passage 3 (Figure 1b). Flow cytometry was used to visualize the expression of stem cell-associated surface markers. The cultured AFSs express predominantly markers of mesenchymal stem cells (*CD44*, *Sca-1*, *CD29*, *CD49f*, and *CD90*) but lack marker expression of the hematopoietic compartment (*CD31 *and *CD45*) (Figure 1c). Furthermore, our cultured murine AFSs express *CD117 *to a similar degree as that reported for human AFS lines [8]. Additionally, they express *CD24*, a pan-epithelial marker in the mouse mammary gland [25]. The derived AFSs are epithelial in nature, as shown by their expression of the luminal epithelial marker keratin 18 (*K18*) and the myoepithelial marker keratin 14 (*K14*). At the same time, they express mesenchymal cells markers, (for example, *vimentin *and α-smooth muscle actin (*α-SMA*) and the mesenchymal stem-cell marker *CD44 *(Figure 1d)).

The hallmark of adult stem cells is their ability to produce progeny of different lineages. We confirmed the multipotency of the cultured murine AFS lines by their differentiation capacity into various cell types *in vitro*, after 14 days of exposure to specific induction conditions (Figure 1e). Differentiation of AFSs in adipogenic medium [9] resulted in the formation of lipid vacuoles verified by Oil red O staining. Osteogenic differentiation medium [9] caused the formation of functional osteoblasts characterized by the deposition of mineralized calcium, as shown by Alizarin Red S staining. The differentiation of AFSs in epithelial medium [26] resulted in the enhancement of the epithelial keratin expression, as shown by Ayoub Shklar staining, a modified Mallory connective tissue stain for keratins [22].

### AFSs closely associate with mammary epithelial cells in the formation of acini in 3D culture

**Figure 2:**
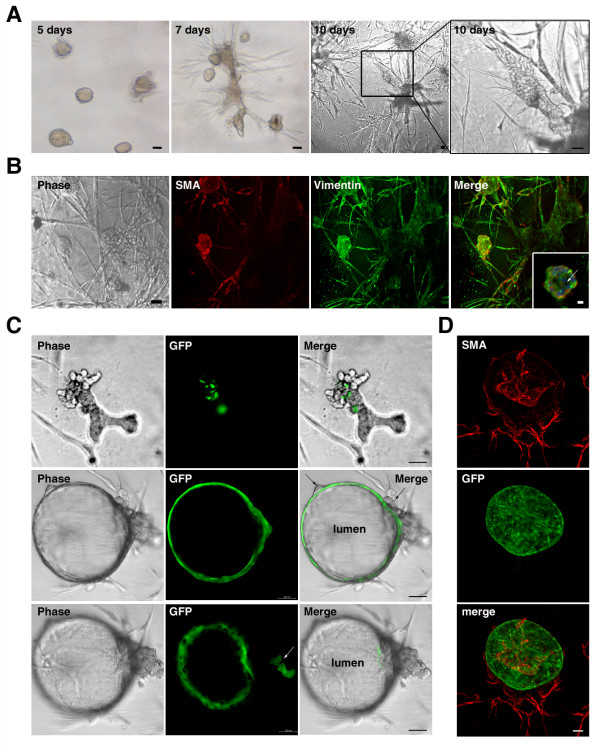
**Amniotic fluid stem cells (AFSs) form spheres and tubular structures in 3D cultures**. AFSs alone or equal numbers of AFSs and MECs derived from GFP transgenic C57/B6-TG (ACTB-EGFP) mice were subjected to 3D differentiation and monitored for up to 10 days after plating. **(a) **AFS-derived progeny form spherical structures 5 days after plating and extend to a branched tubular network in 3D Matrigel culture. **(b) **These structures are positive for *α-SMA *and *vimentin*. Inset shows a section through a spherical structure. **(c) **We observed the emergence of tubular structures formed by AFSs surrounding GFP^+ ^MECs (upper panel) or spheroids formed from GFP^+ ^MECs with a hollow central lumen surrounded by a layer of cells contributed by AFSs (middle panel) and side branches surrounding GFP^+ ^MECs (lower panel). **(d) **AFSs closely associated with GFP^+ ^MECs express α-*SMA*. Scale bars, 50 μm, and for inset, 30 μm.

The interaction of AFSs with primary mammary epithelial cells (MECs) was tested in co-culture experiments. AFSs derived from C57BL/6-Tg (ACTB-EGFP) donors were either mixed with single-cell suspensions of MECs isolated from Rosa26 mice before plating or added to adherent MECs organoid cultures. Interestingly, MECs formed islands after plating surrounded by GFP^+ ^AFS or GFP^+ ^AFS were found in close association with the organoids (data not shown). We extended these observations and performed three-dimensional culture (3D) experiments in the Matrigel microenvironment. MECs display distinct morphogenesis in 3D cultures; they resemble features of the mammary gland architecture *in vivo *[27]. When grown in the presence of basement membrane components, MECs form hollow spherical, acini-like structures with apicobasal polarization formed by a single layer of polarized, growth-arrested cells [23]. AFS lines derived from Rosa26 donors grown under the same conditions in the 3D Matrigel culture system begin to form spherical structures within 5 days of culture, which extend to form tubular networks after 10 days (Figure 2a). This is comparable to the cordlike structures obtained from human AFSs, pre-differentiated toward the endothelial lineage [28]. Immunostaining of the tubular structures showed the expression of the mesenchymal markers vimentin and *α-SMA *(Figure 2b). Closer investigation of the spherical structures revealed the formation of a lumen after 10 days (Figure 2b, inset).

We investigated whether AFSs can associate or cooperate with MECs and integrate into acinar structures formed by MECs. Equal numbers of AFSs, derived from Rosa26 donors, and MECs, derived from GFP transgenic donors, C57BL/6-Tg (ACTB-EGFP), were cultured under conditions that allow 3D differentiation. The formation of acini was monitored 10 days after plating (Figure 2c). We observed the emergence of branching tubular structures formed by AFSs surrounding small clusters of GFP^+ ^MECs (Figure 2c, upper panel) or spheres formed by GFP^+ ^MECs with a hollow central lumen surrounded by a layer of cells contributed by AFS (Figure 2c, middle panel). The AFSs branched off the acini and surrounded additional GFP^+ ^MECs (Figure 2c, lower panel). Immunostaining experiments of the obtained structures with *α-SMA *suggest that AFSs closely associate with MECs and contribute *α-SMA*^+ ^mesenchymal cells to emerging acini (Figure 2d) but do not integrate into the MEC cell layer.

### The microenvironment of the mammary fat pad triggers AFS differentiation into mesenchymal cells

**Figure 3:**
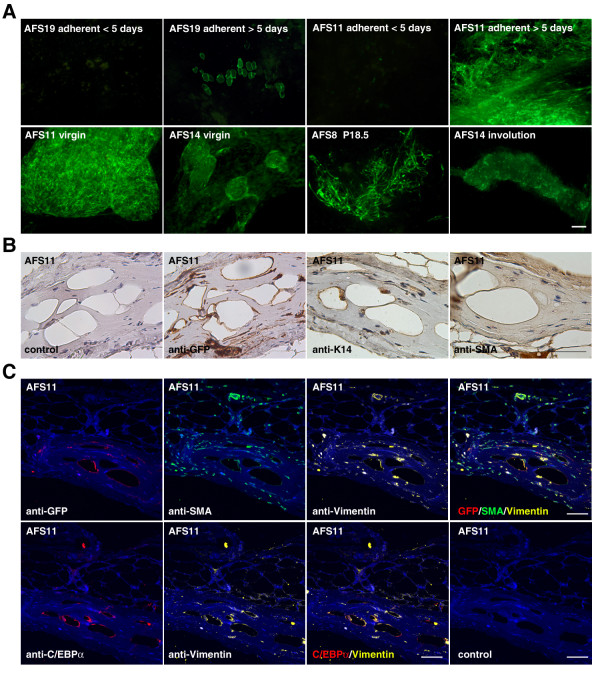
**Cell-fate tracing of transplanted amniotic fluid stem cells (AFSs) into cleared fat pads**. **(a) **The 2 × 10^5 ^AF-derived cells (rapidly adherent within 5 days) and AFSs (slowly adherent, after 5 days of culture) derived from various donors were transplanted into 3-week-old recipient mice and analyzed 8 weeks later as whole mounts. Only the slowly adhering cells selected in the second culture step are able to engraft and can be classified as AFSs. **(b) **Histologic analysis confirms that the GFP^+ ^AFSs survived and differentiated into adipocytes and connective tissue. Scale bar for whole mounts and tissue sections is 50 μm, (Blue) X-Gal stain or hematoxylin, (Pink) Nuclear fast red, and (Brown) DAB staining.

Mammary epithelial outgrowths can be observed in cleared mammary fat pads transplanted with ductal fragments or dispersed mammary cells. This procedure can be used to identify mammary stem cells and to study their properties. We used the fat-pad microenvironment to investigate the regenerative potential of AFS lines derived from GFP-transgenic mice, C57BL/6-Tg (ACTB-EGFP). We compared amniotic fluid cells (AFs) that became adherent within the initial culture period of 5 days and AFS that became adherent after only 5 days of culture. AF cells and AFSs (2 × 10^5 ^each) were inoculated into epithelium-divested inguinal mammary fat pads of 3-week-old recipient Rag 2^-/- ^female mice, and outgrowths were analyzed 8 weeks later with fluorescence microscopy of whole mounts. Only AFSs that became adherent after day 5 of the isolation procedure were able to implant and grow in cleared mammary fat pads (Figure 3a, top panel). The GFP^+ ^outgrowths derived from four independent transplantation experiments with different GFP^+ ^AFS lines yielded mainly connective tissue and contained a mixture of stromal cells and adipocytes (Figure 3a, lower panel). Differentiation into mammary epithelium was not detected. The AFS differentiation capacity was not influenced by pregnancy hormones or tissue remodeling during involution. This observation was confirmed with immunohistochemistry on sections derived from the outgrowths with antibodies specific for GFP, *K14*, and *α-SMA *(Figure 3b). *K14 *and *α-SMA *expression colocalized with the expression of GFP. The GFP^+ ^cells originated from the transplanted AFSs and provide connective tissue components composed of stromal cells and adipocytes. Immunohistochemical analysis showed that the cells originating from the amniotic fluid stem cells (GFP^+^) expressed stromal cell markers (*α-SMA *and *vimentin*) and adipocyte marker (*C/EBPα*) (Figure 3c).

**Figure 4:**
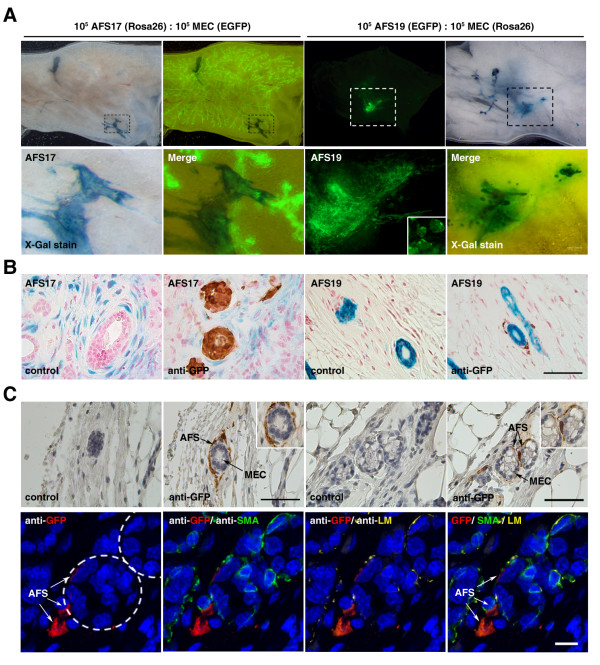
**Cell-fate tracing of amniotic fluid stem cells (AFSs) in co-transplantation with MECs into cleared fat pads**. **(a) **AFSs were transplanted in a 1:1 ratio with supporting MECs (10^5 ^each) and analyzed 8 weeks later. X-Gal-stained whole mounts from virgin mice show that AFS-derived progeny surround the developing ductal structures. **(b) **Histologic analysis of whole mounts inoculated with AFSs and MECs confirms that AFSs form a stromal cell layer around the ductal outgrowth but do not integrate into the myoepithelial or luminal cell layer in virgin mice. **(c) **Histologic analysis of whole mounts at pregnancy day 18.5 inoculated with GFP^+ ^AFSs and MECs shows that the stromal cell layer derived from GFP^+ ^AFSs exhibit no adverse effects on alveologenesis. Insets show higher magnification. Scale bar for whole mounts and tissue sections is 50 μm. A few *α-SMA*^+ ^and GFP^+ ^AFS-derived cells can be detected within the alveolar unit, as shown by *laminin β1 *staining. The circles indicate alveolar units. Scale bar is 10 mm. (Blue) X-Gal stain or hematoxylin, (Pink) Nuclear fast red, and (Brown) DAB staining.

We also examined the possibility that MECs might be required for the participation of AFSs in ductal reconstitution, analogous to the observations made with the transplantation of neural stem cells [21]. It is conceivable that the co-transplantation of mammary epithelial cells can alter the signals acting on AFSs. Equal numbers of MECs and AFS were combined (10^5 ^each) and immediately inoculated into cleared fat pads of 3-week-old Rag 2^-/- ^recipients. Both combinations (AFSs derived from Rosa26 donors combined with GFP^+ ^MECs and AFS derived from C57BL/6-Tg (ACTB-EGFP) donors combined with LacZ^+ ^MECs) were analyzed in virgin, pregnant, and postlactation mice (Figure 4). Eight weeks after transplantation, mice were either mated or analyzed as virgin mice. Whole mounts of the resulting glands were subjected to fluorescence microscopy before staining with X-Gal. The AFS progeny contributed ringlike structures around the growing ducts (Figure 4a), reminiscent of the results obtained in the 3D-culture experiments in virgin mice. Histologic analysis revealed that AFS-derived cells contributed stromal components surrounding the mammary epithelial ducts in virgin mice, but did not incorporate into the epithelial cell layers (Figure 4b). The differentiation stimulus during pregnancy did not alter the cell fate of the majority of transplanted AFSs (Figure 4c). They contributed GFP^+ ^stromal components tightly associated with emerging alveoli (Figure 4c, upper panel). Histologic analysis by using IHC-IF and antibodies specific for GFP, *α-SMA*, and *laminin β1 *revealed that a small number of GFP^+ ^AFSs can be detected associated with alveolar structures (Figure 4c, lower panel). Thus AFS progeny do not incorporate into ductal structures in virgin mice, but can be found in acinar structures during pregnancy. The engrafted AFSs persisted during involution (data not shown).

### AFSs inoculated into the mammary fat pads exhibit a remarkable migratory potential

**Figure 5:**
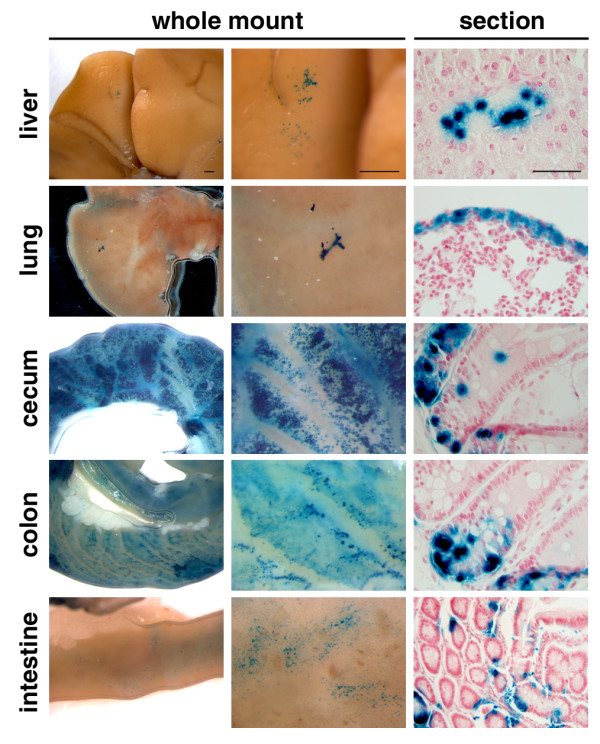
**Amniotic fluid stem cells (AFS)-derived cells migrate to other organs**. The 10^5 ^AFSs derived from a Rosa26 donor were injected into cleared fat pads of 3-week-old recipient mice and analyzed 14 weeks later. X-Gal-stained whole mounts demonstrate the presence of LacZ^+ ^cells in several organs. Tissue sections show the localization of AFS-derived LacZ^+ ^cells between hepatocytes, adjacent to airway epithelial cells, and in the mucosa of the gastrointestinal tract. Scale bar for whole mounts is 500 μm, and for sections, 50 μm, (Blue) X-Gal stain and (Pink) Nuclear fast red staining.

Previous studies indicated that transplanted AFSs can migrate away from the injection site [29], and, if injected intraperitoneally or intravenously, are able to diffuse systemically and to integrate into various tissues [30,31]. We investigated whether AFSs inoculated into the cleared mammary fat pads are able to migrate to other organs. AFSs obtained from Rosa26 mice express the *LacZ *gene, and their progeny can be monitored by whole-mount staining of different organs. On injection of these cells into the mammary fat pad, LacZ^+ ^progeny of AFSs were detected in several organs. The cells inoculated into the number 4 fat pad migrated to the number 3 fat pad on the same side and also were found in healing suture wounds (data not shown). The gastrointestinal tract contained the largest number of LacZ^+ ^cells, followed by the liver and the lung. The AFS-derived cells integrated into the normal tissue architecture (Figure 5).

### The AFSs contribute stromal components to emerging ducts

**Figure 6:**
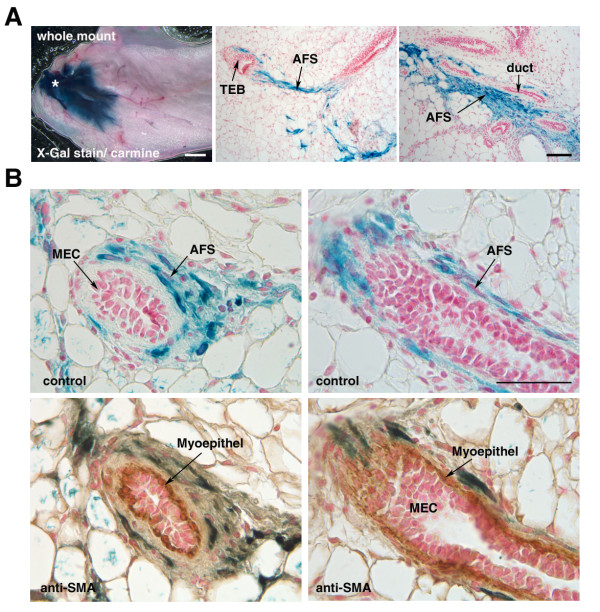
**Amniotic fluid stem cells (AFS)-derived cells contribute stromal components for extending ducts**. The 10^5 ^AFSs derived from a Rosa26 donor were injected into noncleared fat pads of 3-week-old recipient mice and analyzed 2 weeks later. **(a) **Whole-mount analysis and tissue sections of X-Gal-stained whole mounts demonstrate the presence of LacZ^+ ^cells in the stroma of extending ducts. **(b) **AFS-derived LacZ^+ ^cells retain the expression of *α-SMA *but did not integrate into the myoepithelial cell layer. Scale bar for whole mount is 1,000 mm, and for tissue sections, 50 μm; (*) injection site, (Blue) X-Gal stain, (Pink) Carmine alum or Nuclear fast red staining and (Brown) DAB staining.

To investigate whether the AFSs can contribute mesodermal components to endogenous epithelium, we injected 10^5 ^AFSs derived from a Rosa26 donor into noncleared fat pads of 3-week-old Rag 2^-/- ^recipients in close proximity to the nipple (Figure 6). Glands were excised 2 weeks later, subjected to X-Gal staining, and counterstained with carmine alum to visualize the ductal epithelium. Whole-mount analysis demonstrates engraftment and massive proliferation of the transplanted AFSs (Figure 6a). The LacZ^+ ^cells fill the fat pad around the injection site and surround the endogenous, elongating ducts, reminiscent of the co-transplantation experiments with exogenously added MECs. Immunohistochemistry of paraffin sections confirmed the presence of LacZ^+ ^cells in the stromal compartment of the extending endogenous ducts and around terminal end buds (TEBs), but also revealed areas of adipocyte differentiation. The LacZ^+ ^cells expressed the myoepithelial marker *α-SMA*, but did not incorporate into the myoepithelial cell layer (Figure 6b). Based on these observations, we conclude that the stimulus and signals provided by the fat pad only trigger differentiation programs of AFS into mesenchymal cells types, despite their original, partially epithelial nature.

## Discussion

Amniotic fluid-derived stem cells (AFSs) are a most versatile cell population and are interesting for conceptual and practical purposes. Conceptually, they can be used to investigate the culture conditions and the molecular signals required for stem cells to assume particular cell fates. Practically, AFS, triggered by defined cues to differentiate into certain cell types, can potentially be used in transplantation studies for tissue repair or tissue reconstitution. Despite their obvious potential value, a routine procedure has yet to be established for the derivation of AFSs, their growth in culture, and the maintenance of their pluripotent nature.

Several protocols differ considerably from each other. A single-step cultivation method has been used in which AFSs are obtained after 7 days of cultivation without medium changes [29,32-34]. A two-stage culture method favors the isolation of *Oct-4*-positive AFSs [9] and the isolation of *CD117*-positive AFSs [8,30]. These methods use different growth media and isolation methods and yield AFSs of distinct phenotypes. In our experiments, we made use of the two-stage culture method developed for the isolation and enrichment of human AFSs obtained by amniocentesis for prenatal diagnosis [9] and applied this method to the investigation of murine AFSs. During this procedure, AFSs slowly adapt to adherent growth and can be classified according to their morphologic appearance. The cells maintain their ability to differentiate into adipogenic, osteogenic, myogenic, endothelial, neurogenic, and hepatic cells *in vitro *[8,9]. We consistently observed that the amniotic fluid-derived stem cells became adherent between days 5 and 7 after plating. These cells showed unlimited proliferation and the maintenance of their differentiation potential. Conversely, the amniotic fluid cells that became adherent within the first 5 days after plating underwent rapid growth senescence and did not engraft in cleared mammary fat pads, irrespective of the presence of supporting mammary epithelial cells.

The majority of the murine AFSs used in this study appeared fibroblastoid, proliferated rapidly, and co-expressed keratins and vimentin, confirming their partial epithelial nature. This is in contrast to bone marrow-derived mesenchymal stem cells (BM-MSCs), which need special medium supplements to differentiate toward the epithelial lineage [26] and proliferate at a lower rate than AFSs [35]. The multipotency of the murine AFSs was confirmed by *in vitro *differentiation assays. Adipogenic, osteogenic, and epithelial lineages were observed.

In this study, we investigated whether the signals provided by the mammary microenvironment are sufficient to redirect the cell fate of multipotent AFSs into mammary epithelium. We also probed the influence of exogenously added mammary epithelial cells on the differentiation potential of AFS in both *in vitro *and *in vivo *assays. We investigated the *in vitro *morphogenesis of AFSs in a 3D-on-top culture system, a test assay to monitor mammary acinar architecture [23]. In this assay system, we observed the formation of spherelike structures with a small lumen by AFSs followed by the emergence of branched tubular structures that mimic the sprouting of predifferentiated AFSs toward endothelium [28] or endothelial precursor cells [36]. Interestingly, the 3D coculture of AFSs and MECs yielded similar results. MECs were embedded in tubes formed by AFS or developed acinar structures surrounded by a layer of AFSs extending away from the spherical structures. It appears that the AFSs provide stromal components to the glandular epithelium and form a scaffold around MECs.

We also analyzed the differentiation potential of AFSs *in vivo *by transplantation experiments into cleared and noncleared mammary fat pads. Previous studies using cells from seminiferous tubules [20] or neural stem cells [21] of male WAP-Cre/Rosa26 reporter mice showed that the mammary microenvironment could alter the intrinsic cell fate of exogenously provided stem cells toward the mammary epithelial cell lineage after pregnancy. Mesenchymal stem cells from different sources have the potential to differentiate into a variety of cells under the influence of certain microenvironments. Despite the partial epithelial nature of the cultured AFSs, we consistently observed that the progeny of AFSs adopt mesenchymal cell properties after transplantation into the mammary microenvironment. This is in contrast to the engraftment capacity of cells from seminiferous tubules or neural stem cells, which did not persist in the absence of supporting mammary epithelial cells [20,21]. The mammary fat pad is of mesodermal origin and provides factors that cause AFS differentiation toward mesenchymal cell types. The morphogenetic capacity of the ectoderm-derived mammary epithelium strongly depends on the microenvironmental clues provided by the fat pad. It is, therefore, interesting to note that AFSs contribute stromal components to emerging ducts that have no adverse effect on mammary epithelial repopulation capacity. The engrafted AFSs continued to express myoepithelial markers but did not integrate into the luminal or myoepithelial layer of the emerging ducts in virgin mice.

Myoepithelial cells play an important role in the morphogenetic events during gland development and influence the proliferation, survival, and differentiation of luminal cells and modulate stromal-epithelial interactions [37]. Interestingly, we observed the presence of a small number of AFS-derived cells in close proximity to emerging alveoli during pregnancy.

At this point, we cannot distinguish between two possible interpretations. It is conceivable that AFSs are intrinsically limited in their differentiation potential, that they are unable to assume the mammary epithelial cell fate, and that the stroma-induced transdifferentiation potential of fetal or adult tissue might be restricted to cell fates of the same germ layer. For example, endoderm-derived pulmonary and pancreatic epithelia failed to exhibit morphogenetic responses to the adult mammary fat pad [38]. It is possible that our AFS lines emerged from a germ-layer lineage that is not conducive to differentiate into epithelium in response to the signals provided by the mammary fat pad, even in the presence of MECs. In concordance with this notion is the observation that neural stem cells [21] are able to differentiate into mammary epithelium, but only after cotransplantation with MECs depleted of endogenous stem cells and after the hormonal stimulus exerted during pregnancy. These cells are derived from the same germ layer as the mammary gland, the ectoderm that might account for this differentiation potential.

Alternatively, it is possible that the AFSs are not exposed to the proper signals required for mammary epithelial differentiation. The timing of the exposure and the cellular environment might contribute influences that we cannot control at present. This might explain the presence of a small number of AFS-derived cells within an alveolar unit during pregnancy. Interestingly, transplantation under the renal capsule indicates that mammary mesenchyme can be replaced by salivary [38] or prostatic [39] mesenchyme to facilitate mammary epithelial morphogenesis. Based on these observations, it is conceivable that the AFS-derived mesenchyme supports mammary epithelial morphogenesis. In contrast to cleared mammary fat pads, the renal capsule is a highly vasculogenic graft site that supports the engraftment of human mammary tissue and other heterologous tissue components. For this reason, it has been used in many tissue-transplantation studies [40].

The results obtained in our *in vivo *studies are consistent with the observations we made in the *in vitro *experiments. AFSs participate in the ductal outgrowth, persist over long periods in association with the luminal and myoepithelial cells, but do not become part of the ductal epithelial structures. They contribute mesenchymal components to the glandular epithelium and are remarkably motile. Because of this motile phenotype, it is not too surprising that AFS-derived cells are occasionally incorporated into mammary epithelial structures during pregnancy. The inoculated AFSs proliferate, and their progeny can be detected in a number of distant organs several weeks after transplantation. LacZ^+ ^cells were found in other epithelial tissue, such as the lung and the mucosa of the gastrointestinal tract. LacZ^+ ^cells at these sites were incorporated into the tissues. Previous reports have shown that BM-MSCs and AFS can contribute to airway-epithelium restoration [30,31,41], and BM-MSCs migrate and participate in gastrointestinal tract mucosa regeneration after damage [42]. The ability of AFSs to migrate and engraft at distant sites and to incorporate into the gastrointestinal mucosa suggests that the signals of the microenvironment can influence the cell fate of AFSs.

## Conclusions

The observation that undifferentiated AFSs survive in a permissive environment and have the potential to differentiate into multiple cell types makes them interesting candidates for use as therapeutic cells and for their application in tissue repair. Here we demonstrated that the signals provided by the mammary microenvironment and the communication between AFSs and mammary epithelium favor the formation of supportive stroma for the extending ductal epithelium, but do not cause the epithelial differentiation of AFSs. AFSs exhibit a remarkably motile phenotype, and their ability to migrate from the mammary fat pad to other organs might shed light on mechanisms of metastasis formation.

## Abbreviations

*α-SMA*: Alpha smooth muscle actin; AFSs: amniotic fluid stem cells; BM-MSCs: bone marrow-derived mesenchymal stem cells; MECs: mammary epithelial cells; TEB: terminal end bud.

## Competing interests

The authors declare that they have no competing interests.

## Authors' contributions

PK developed ideas, designed and conducted the experiments, and wrote and edited the manuscript. VV conducted and contributed to the analysis of data and edited the manuscript. BG developed ideas and edited the manuscript.
